# Effects of short‐chain acyl‐CoA dehydrogenase on cardiomyocyte apoptosis

**DOI:** 10.1111/jcmm.12828

**Published:** 2016-03-17

**Authors:** Zhenhua Zeng, Qiuju Huang, Zhaohui Shu, Peiqing Liu, Shaorui Chen, Xuediao Pan, Linquan Zang, Sigui Zhou

**Affiliations:** ^1^ Department of Clinical PharmacyGuangDong Pharmaceutical UniversityGuangzhouChina; ^2^ Department of Pharmacology and ToxicologySchool of Pharmaceutical SciencesSun Yat‐sen UniversityGuangzhouChina

**Keywords:** short‐chain acyl‐CoA dehydrogenase, cardiomyocyte apoptosis, energy metabolism•, tert‐butyl hydroperoxide, peroxisome proliferator‐activated receptor α, AMP‐activated protein kinase

## Abstract

Short‐chain acyl‐CoA dehydrogenase (SCAD), a key enzyme of fatty acid β‐oxidation, plays an important role in cardiac hypertrophy. However, its effect on the cardiomyocyte apoptosis remains unknown. We aimed to determine the role of SCAD in tert‐butyl hydroperoxide (tBHP)‐induced cardiomyocyte apoptosis. The mRNA and protein expression of SCAD were significantly down‐regulated in the cardiomyocyte apoptosis model. Inhibition of SCAD with siRNA‐1186 significantly decreased SCAD expression, enzyme activity and ATP content, but obviously increased the content of free fatty acids. Meanwhile, SCAD siRNA treatment triggered the same apoptosis as cardiomyocytes treated with tBHP, such as the increase in cell apoptotic rate, the activation of caspase3 and the decrease in the Bcl‐2/Bax ratio, which showed that SCAD may play an important role in primary cardiomyocyte apoptosis. The changes of phosphonate AMP‐activated protein kinase α (p‐AMPKα) and Peroxisome proliferator‐activated receptor α (PPARα) in cardiomyocyte apoptosis were consistent with that of SCAD. Furthermore, PPARα activator fenofibrate and AMPKα activator AICAR treatment significantly increased the expression of SCAD and inhibited cardiomyocyte apoptosis. In conclusion, for the first time our findings directly demonstrated that SCAD may be as a new target to prevent cardiomyocyte apoptosis through the AMPK/PPARα/SCAD signal pathways.

## Introduction

Cardiovascular disease shows the relatively higher levels of morbidity and mortality all over the world. Heart failure is the terminal stage of various kinds of cardiovascular diseases. Large amount of evidence suggested that cardiomyocyte apoptosis played a critical role in the development of heart and numerous cardiac diseases 1, 2. Moreover, cardiomyocyte apoptosis is a necessary process of transition from pathological cardiac hypertrophy to heart failure 3, 4, 5.

As described earlier, the unbalance of energy metabolism was involved in the myocardial apoptosis 6. Excess energy was consumed in heart than any other organs, while 60–90% was supplied from fatty acid β‐oxidation 7. While, in the failing heart, cardiac substrate utilization and energy metabolism altered, were known as ‘metabolic remodelling’. Short‐chain acyl‐CoA dehydrogenase (SCAD) is a member of acyl‐CoA dehydrogenases family, which catalyses the first step in mitochondrial β‐oxidation of fatty acids 8. Our previous researches had revealed that SCAD expression was significantly down‐regulated in pathological cardiac hypertrophy *in vivo* and *in vitro* for the first time. Moreover, we also have found that pathological cardiac hypertrophy exhibited SCAD changes in myocardial fatty acids utilization 9, 10. Nevertheless, the role of SCAD in cardiomyocyte apoptosis is currently still unknown.

Peroxisome proliferator‐activated receptor‐α (PPARα), a member of fatty acid‐activated nuclear receptor family, was involved in the development of left ventrical hypertrophy and cardiomyocyte apoptosis 11, 12. Previous studies have shown that SCAD gene was regulated by PPARα 10, 13, 14. AMP‐activated protein kinase (AMPK) is a crucial metabolic energy sensor and its activation has been reported to directly reflect with the heart against hypertrophy, ischaemic injury and cell death 15. AMPK and PPARα is involved in the inhibition of gluconeogensis and fatty acid oxidation 16. It has been shown that AMPK activation inhibited cardiac hypertrophy through the reactivation of PPARα signalling pathway 17. Besides, we have previously shown that deactivation of PPARα led to the decrease in SCAD expression in pathological cardiac hypertrophy 10. However, the role of the AMPK/PPARα pathway in the prevention of cell apoptosis induced by acute oxidative stress remains unclear.

Therefore, this study was designed to investigate the effects of SCAD on tBHP‐induced cardiomyocyte apoptosis, and explore the regulation of AMPK/PPARα/SCAD signalling pathways on cardiomyocyte apoptosis.

## Materials and methods

### Primary cultures of neonatal rat cardiomyocytes

Primary cultures of neonatal rat cardiomyocytes (NRCMs) were prepared with the method previously described 13. Two‐ to three‐day‐old Sprague–Dawley rats were obtained from the Laboratory Animal Center of Guangzhou University of Chinese Medicine. Experiments were approved by the Institutional Animal Care and Use Committee of GuangDong Pharmaceutical University. In brief, 2–3‐day‐old Sprague–Dawley rats were killed with ethyl ether. The hearts were surgically removed from the animals, minced into 1–3‐mm^3^ pieces, and then centrifuged twice instantly with PBS at 500 × g. Prior to trypsinization, the minced tissue was treated with 0.08% trypsin solution on ice for 20 min. with discontinuous shaking for better mixing. Following the pre‐cooling step, the tissue was subjected to 3–4 cycles of proteolytic dissociation by magnetic stirring (10 min, 37°C) with trypsin solution, which contained 0.05 mg/ml DNase I from the second cycle. Supernatants from each cycle were pooled and centrifuged. In the end, the cell pellet was resuspended in DMEM supplemented with 20% calf serum, 100 U/ml penicillin and 100 mg/ml streptomycin. Selective adhesion procedure was performed after a 1.5‐hrs incubation at 37°C in a humidified atmosphere (5% CO_2_ and 95% air) to obtain a high purity of the cardiomyocyte population. Subsequently, 0.1 mM bromodeoxyuridine was added to the medium for the first 48 hrs of culture. More than 95% of cells were cardiomyocytes, as demonstrated by immunostaining with α‐sarcomeric actin antibody. The medium was then changed to serum‐free medium containing 1% bovine serum albumin, 50 U/ml penicillin and 50 mg/ml streptomycin 12 hrs prior to further treatments.

### Cell viability assay

Cells were seeded in a 96‐well culture plate. Cell viability were determined with 3‐[4,5‐dimethylthiazol‐2‐yl]‐2,5‐diphenyl‐tetrazolium (MTT) reduction assay according to the manufacturer's instructions. The culture medium was removed and the cells was dissolved in dimethylsulfoxide and shaken for 15 min. The absorbance was determined by the microplate reader with 570 nm.

### RNA interference

siRNA‐1186, the most efficient small interference RNA (siRNA) for SCAD, was used for our experiments 10. The sequence of siRNA‐1186 were: sense 5′‐CCGCAUCACUGAGAUCAUTT‐3′ and antisense 5′‐AUAGAUCUCAGUGAU GCGGTT‐3′. After cultured for 24 hrs, cardiomyocytes were transfected with 100 nM siRNA‐1186 or negative control using Lipofectamine 2000. After transfection for 72 hrs, the NRCMs were harvested for next experiments.

### Quantitative real‐time polymerase chain reaction

Total RNA of cultured NRCMs was extracted with Trizol reagent (TaKaRa, Shiga, Japan). Reverse transcription was performed at 37°C for 15 min., and 85°C for 5 sec., using the PrimeScript^™^ RT reagent kit with gDNA Eraser(TaKaRa, Shiga, Japan). The SCAD mRNA was determined by using SYBR^®^ Premix ExTaq^™^ kit (TaKaRa, Shiga, Japan). GADPH was served as an endogenous control. The comparative 2^−ΔΔCt^ method was used to calculate the expression of mRNA 18. The primer sequences were as shown in Table 1.

**Table 1:** The primers for the real‐time PCR amplification

Gene	The primer sequence (5′–3′)
SCAD	Forward: TGCCCTATGTTTCGCACCTC Reverse: TTCAATGCCCATCATCCCTT
PPARα	Forward: CCTGGCAATGCACTGAACATC Reverse: ACGCCGTTGGCTACCATCTTG
GADPH	Forward: AGGAGTAAGAAACCCTGGAC Reverse: CTGGGATGGAATTGTGAG

### Western blot analysis

Western blot analysis of total protein isolated from NRCMs was performed as previously described 13. Briefly, protein samples (30 or 50 μg) were separated in 10% and 15% SDS‐PAGE and blotted to PVDF membrane. The membranes were incubated with the primary antibodies including rabbit anti‐SCAD monoclonal antibody (diluted 1:1000; Abcam, Cambridge, UK), anti‐Caspase‐3 polyclonal antibody (diluted 1:1000; Cell Signaling Technology, Danvers, MA, USA), Bcl‐2 polyclonal antibody (diluted 1:1000; Cell Signaling Technology, Danvers, MA, USA), Bax polyclonal antibody (diluted 1:1000; Cell Signaling Technology, Danvers, MA, USA), AMPK monoclonal antibody (diluted 1:1000; Cell Signaling Technology, Danvers, MA, USA), p‐AMPK monoclonal antibody (diluted 1:1000; Cell Signaling Technology, Danvers, MA, USA) and mouse anti‐PPARα monoclonal antibody (diluted 1:500; Sigma‐Aldrich, St.Louis, MO, USA), anti‐α‐tubulin monoclonal antibody (diluted 1:5000; Sigma‐Aldrich, St.Louis, MO, USA), and then the HRP conjugated goat‐antimouse or rabbit IgG1 secondary antibody (1:10,000; Sigma‐Aldrich, St.Louis, MO, USA) were used. Finally, the protein expression levels were detected with chemiluminescent substrate (Thermo, Waltham, MA, USA). Results were analysed with the Image J system.

### Hoechst 33258 nucleus staining

Hoechst 33258 nucleus stainin kit was used to determine the apoptosis. Cells were seeded in 24‐well plates. After treatment, we washed the cells twice in PBS and fixed with 4% formalin for 10 min. We then used 500 ul Hoechest 33258 to stain them for 15 min. Following three washes, we examined the cells with a fluorescence microscope at 200× magnifications.

### Annexin V‐FITC/PI staining assay

Annexin V and PI fluorescein staining kits (KaiJi, Nanjing, China) were utilized to measure cardiomyocyte apoptosis by following manufacturer's instruction. Briefly, after various treatments, the cells were digested with 0.25% trypsin and collected by centrifugation. The cells were then resuspended in 500‐ml binding buffer and finally stained with Annexin V and PI for 20 min. in the dark. The samples were evaluated by Flow Cytometry within 1 hr.

### SCAD enzyme activity assay

Protein was extracted after NRCMs were harvested, and then quantified according to the Pierce^®^ BCA protein assay kit (Thermo, Waltham, MA, USA). Short‐chain acyl‐CoA dehydrogenase enzyme activity was detected according to the cell SCAD assay kit (GenMed, Plymouth, MN,USA).

### Free fatty acid content detection

After being harvested, NRCMs were disrupted using Manual cell disruptor, and then quantified according to Pierce^®^ BCA protein assay kit (Thermo, Waltham, MA, USA). Free fatty acid content was detected using Nonesterified free fatty acids assay kit (Nanjing Jiancheng Bioengineering Institute, China).

### ATP content detection

The cellular ATP levels were determined using an ATP assay kit (Beyotime, Shanghai, China) following the manufacturer's instructions. Cells were lysed with 200 μl cell lysis reagent (Beyotime, Shanghai, China) and then quantified using the Pierce^®^ BCA protein assay kit (Thermo, Waltham, MA, USA). Luciferase reagent (1 μl) and dilution buffer (100 μl) were added to each well of a black 96‐well culture plate. After 3 min., 50 μl samples of lysate were added to the wells. The luminescence was then measured with a luminometer. Total ATP levels were expressed as nmol/mg protein and adjusted to that in the control group.

### Statistical analysis

Data were presented as the mean ± S.D. from at least three independent experiments. The data are analysed using GraphPadPrism 5.0 (La Jolla, CA, USA) program software. Statistical evaluation of the data was performed by one‐way anova followed by a Bonferroni‐corrected Student's *t*‐test for multiple comparisons. *P* < 0.05 was considered significant.

## Results

### Cytotoxic effect of tBHP on NRCMs

**Figure 1:**
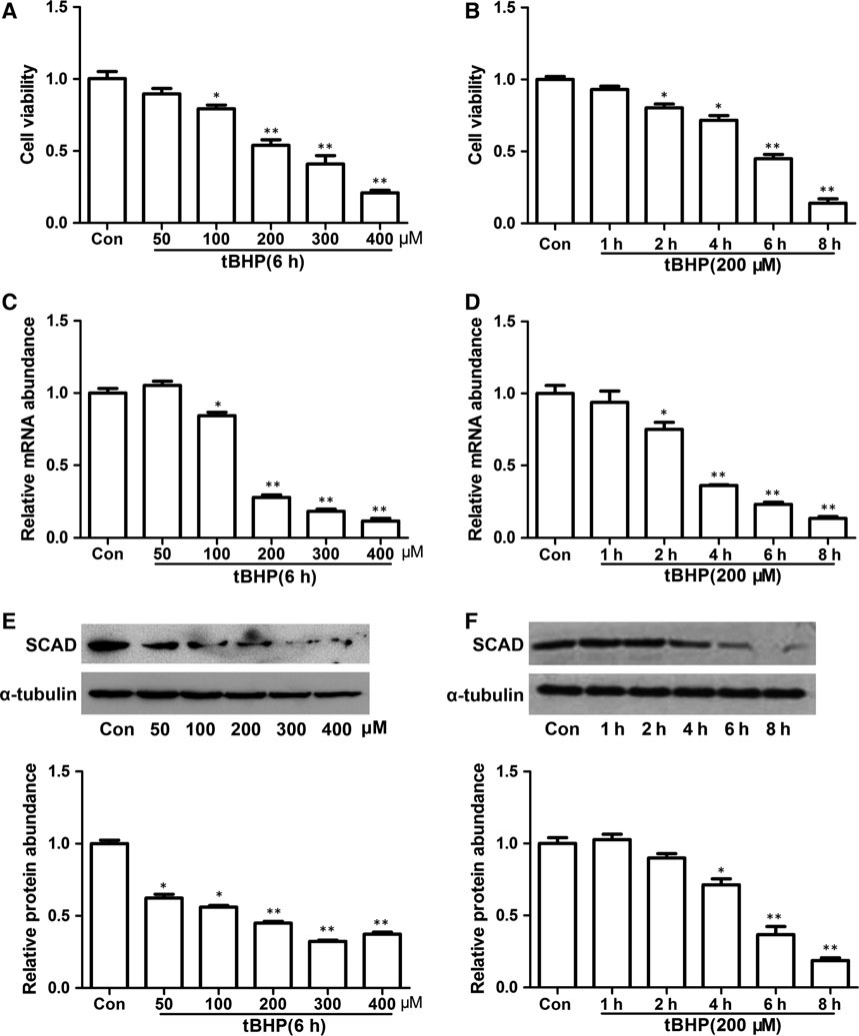
Cell viability and SCAD expression were decreased by tBHP in cardiomyocyte. (**A**) Cardiomyocytes were treated with various concentrations of tBHP for 6 hrs and assessed for cell viability. (**B**) Cardiomyocytes were treated with 200 μM tBHP for different incubation times. The levels of mRNA (**C** and **D**) and protein of SCAD (**E** and **F**) were mearused by real‐time PCR and Western blots. Cardiomyocytes were treated with different concentrations of tBHP for 6 hrs and 200 μM tBHP for different incubation time. Mean ± S.D., *n* = 3, *P < 0.05; **P < 0.01 *versus* control.

The viability of NRCMs was determined using the MTT assay. Neonatal rat cardiomyocytes were treated with different concentrations of tBHP for 6 hrs and different time for 200 μM tBHP. As shown in Figure 1A and B, tBHP decreased cell viability of NRCMs in a dose‐ and time‐dependent manner. Correspondingly, cell viability was about 50% after NRCMs exposed tBHP at 200 μM for 6 hrs. Therefore, we chose 200 μM tBHP, 6 hrs as the favourable conditions for all subsequent experiments.

### SCAD expression was significantly decreased in tBHP‐induced NRCMs

Short‐chain acyl‐CoA dehydrogenase expression in tBHP‐induced NRCMs apoptosis was analysed at both mRNA and protein levels by quantitative real‐time PCR (qRT‐PCR) and Western blot assay. As shown in Figure 1C–F, SCAD mRNA and protein expression were consistently declined when NRCMs were treated with different concentration tBHP for 6 hrs. In addition, we investigated the SCAD expression change in cardiomyocytes treated with 200 μM tBHP for varying time. Results indicated that mRNA and protein expression of SCAD were significantly decreased with 200 μM tBHP for 6 and 8 hrs respectively.

### SCAD played a critical role in cardiomyocyte apoptosis

**Figure 2:**
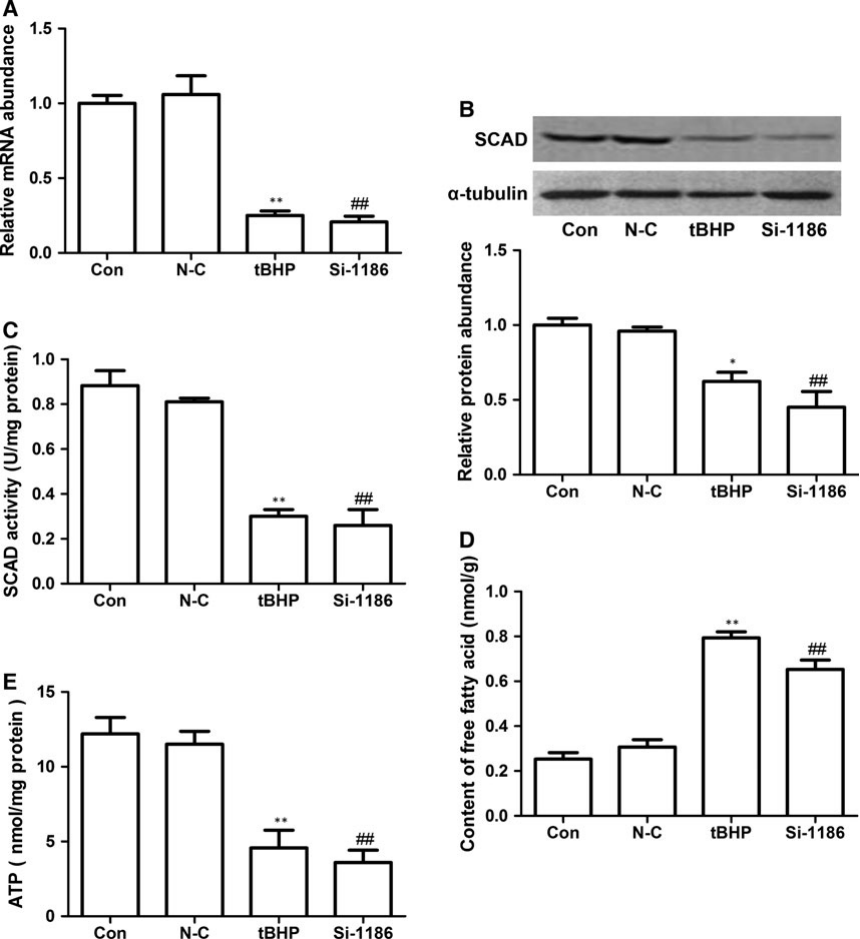
The expression and enzyme activity of SCAD in the cardiomyocytes treated with tBHP (200 μM, 6 hrs) or siRNA‐1186 (72 hrs). (**A**) The relative mRNA level of SCAD was significantly inhibited by siRNA‐1186 and tBHP. (**B**) The relative protein level of SCAD was significantly inhibited by siRNA‐1186 and tBHP. (**C**) The enzyme activity of SCAD was significantly inhibited by siRNA‐1186 and tBHP. (**D**) Content of free fatty acid was significantly increased in cardiomyocytes treated with siRNA‐1186 or tBHP. (**E**) The ATP level was significantly decreased in cardiomyocytes treated with siRNA‐1186 or tBHP. N‐C: negative control siRNA. Mean ± S.D., *n* = 3, *P < 0.05, **P < 0.01 *versus* control. ##*P* < 0.01 *versus* N‐C.

To explore the role of SCAD in NRCMs apoptosis, the technique of RNA interference was employed. siRNA‐1186 was transfected in NRCMs for 72 hrs as we reported previously 10. As shown in Figure 2A–C, compared with control group, the expression and enzyme activity of SCAD were significantly decreased in siRNA‐1186 and tBHP groups. In addition, ATP content was significantly lower in siRNA‐1186‐ and tBHP‐treated NRCMs, however, the content of free fatty acids were obviously increased in the two groups (Fig. 2D and E).

**Figure 3:**
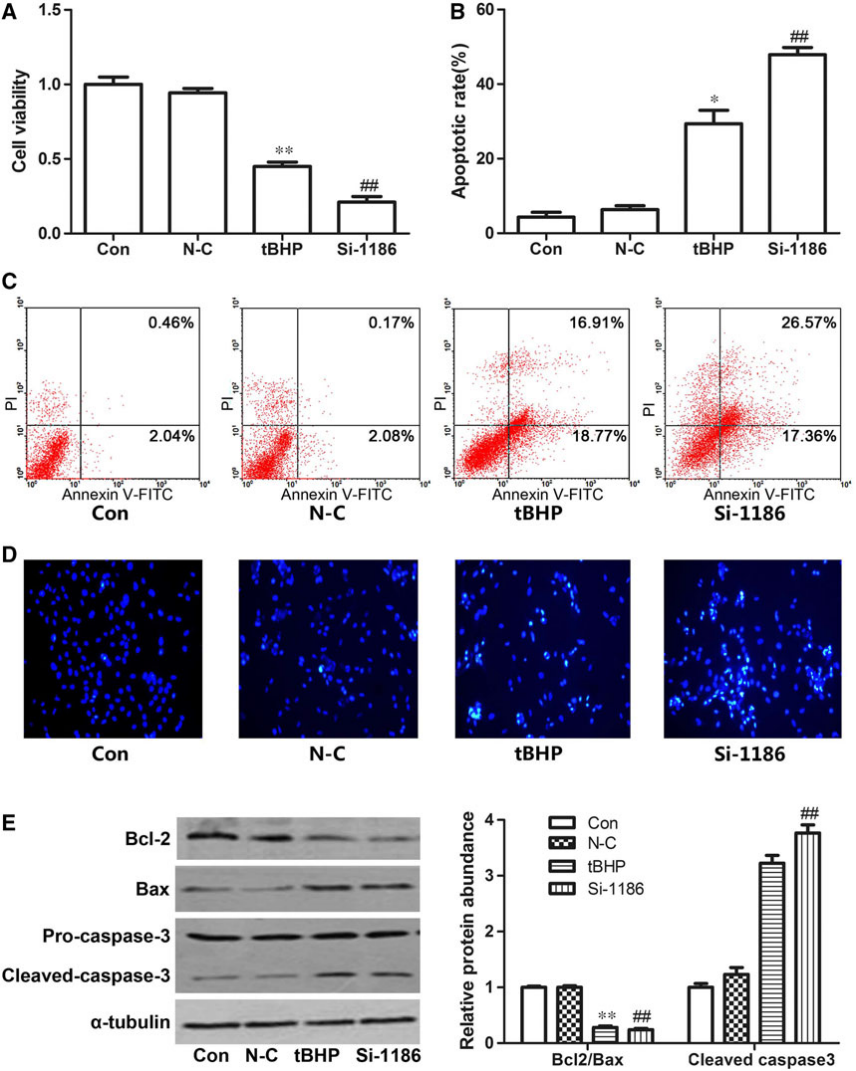
The cell apoptotic in the cardiomyocytes treated with tBHP (200 μM, 6 hrs) or siRNA‐1186 (72 hrs). The changes of cell viability (**A**) and cell apoptotic rate (**B** and **C**) in the cardiomyocytes treated with tBHP (200 μM, 6 hrs) or siRNA‐1186 (72 hrs). (**D**) Cell apoptosis was determined with Hoechst 33258 staining. 200× magnification. (**E**) The expression of apoptosis‐related proteins in tBHP‐ or siRNA‐1186‐induced cardiomyocytes. N‐C: negative control siRNA. Mean ± S.D., *n* = 3, *P < 0.05; **P < 0.01 *versus* control. ##*P* < 0.01 *versus* N‐C.

In siRNA‐1186 and tBHP group, the cell viability was significantly decreased (Fig. 3A). Correspondingly, the cell apoptotic rate was markedly increased by Hoechst 33258 assay and Annexin V‐FITC/PI staining assay (Fig. 3B–D). To investigate the mechanism of cardiomyocyte apoptosis, we examined the protein levels of Bcl‐2, Bax and cleaved caspase3. Western blot analyses showed that Bcl‐2 was decreased in siRNA‐1186 and tBHP groups. On the contrary, Bax and cleaved caspase 3 protein levels were markedly increased in the two groups compared with control group (Fig. 3E). These results indicated that SCAD siRNA treatment triggered the same apoptosis as NRCMs treated with tBHP.

### Effects of PPARα on cardiomyocyte apoptosis

**Figure 4:**
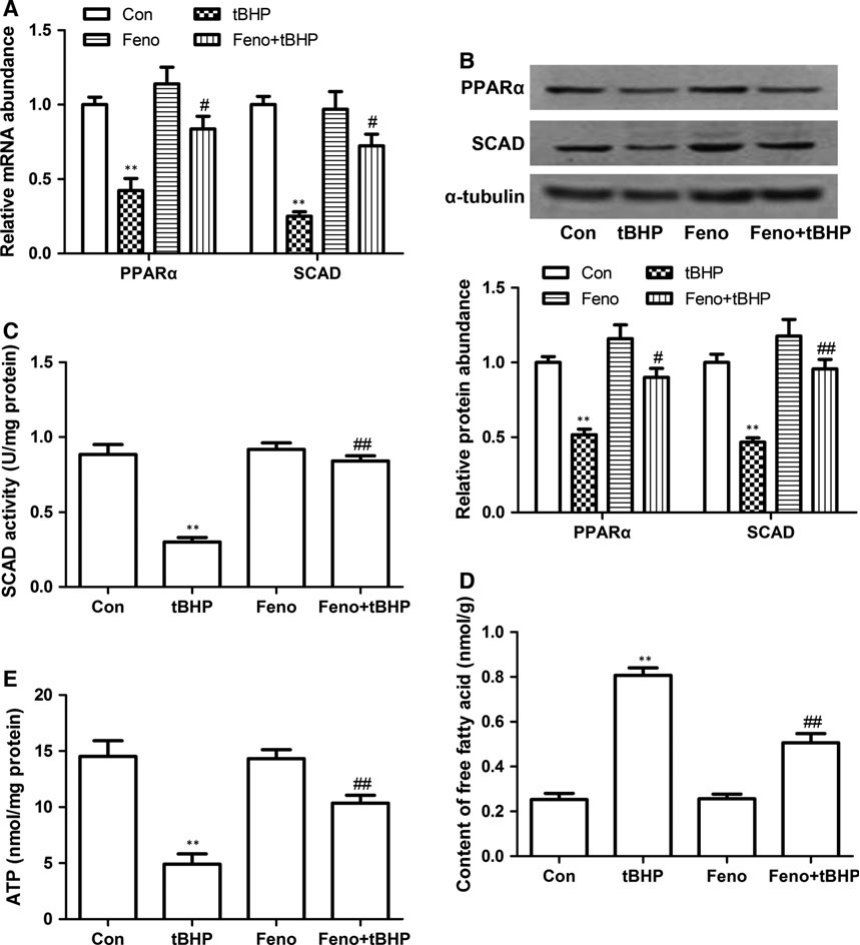
Effects of Feno on cardiomyocyte apoptosis in tBHP‐treated cardiomyocytes. (**A**) The PPARα and SCAD mRNA expression was significantly decreased in cardiomyocytes treated with tBHP, however, increased in cardiomyocytes pre‐treated with Feno. (**B**) The PPARα and SCAD protein expression was significantly decreased in cardiomyocytes treated with tBHP, however, increased in cardiomyocytes pre‐treated with Feno. (**C**) The SCAD enzyme activity was significantly decreased in cardiomyocytes treated with tBHP, however, increased in cardiomyocytes pre‐treated with Feno. (**D**) The content of free fatty acid was significantly increased in cardiomyocytes treated with tBHP, however, decreased in cardiomyocytes pre‐treated with Feno. (**E**) The ATP level was significantly decreased in cardiomyocytes treated with tBHP, however, increased in cardiomyocytes pre‐treated with Feno. Mean ± S.D., *n* = 3, *P < 0.05, **P < 0.01 *versus* control, #*P* < 0.05, ##*P* < 0.01 *versus* tBHP.

**Figure 5:**
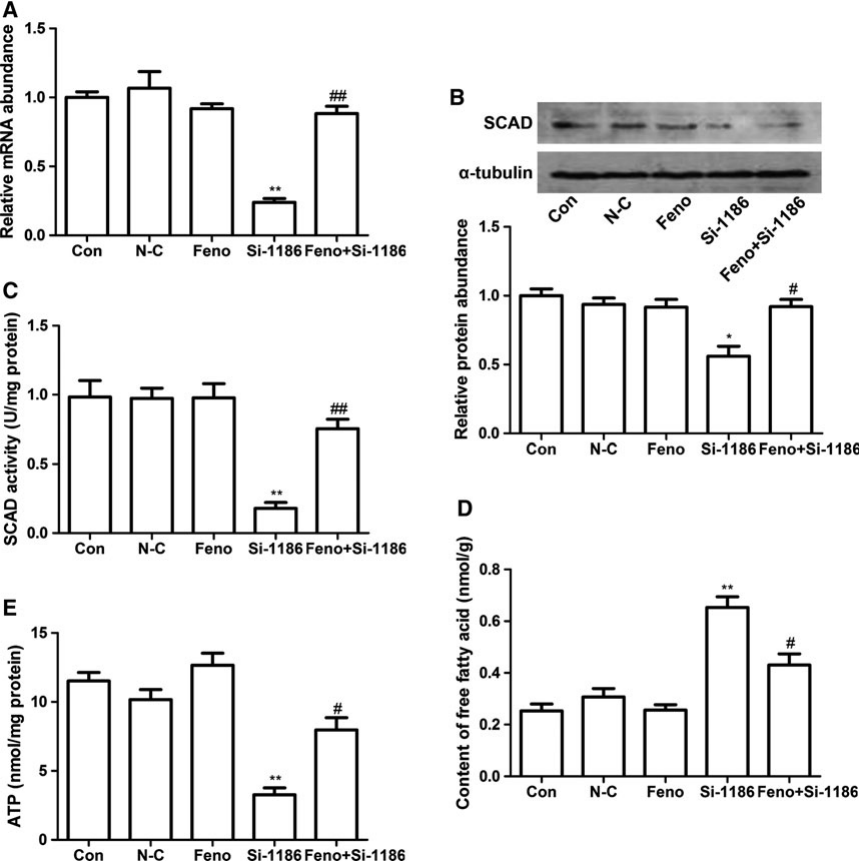
Effects of Feno on cardiomyocyte apoptosis in siRNA‐1186‐treated cardiomyocytes. (**A**) The SCAD mRNA expression was significantly decreased in cardiomyocytes treated with siRNA‐1186, however, increased in cardiomyocytes pre‐treated with Feno. (**B**) The SCAD protein expression was significantly decreased in cardiomyocytes treated with siRNA‐1186, however, increased in cardiomyocytes pre‐treated with Feno. (**C**) The SCAD enzyme activity was significantly decreased in cardiomyocytes treated with siRNA‐1186, however, increased in cardiomyocytes pre‐treated with Feno. (**D**) The content of free fatty acid was significantly increased in cardiomyocytes treated with siRNA‐1186, however, decreased in cardiomyocytes pre‐treated with Feno. (**E**) The ATP level was significantly decreased in cardiomyocytes treated with siRNA‐1186, however, increased in cardiomyocytes pre‐treated with Feno N‐C: negative control siRNA. Mean ± S.D., *n* = 3, *P < 0.05, **P < 0.01 *versus* control, #*P* < 0.05, ##*P* < 0.01 *versus* si‐1186.

**Figure 6:**
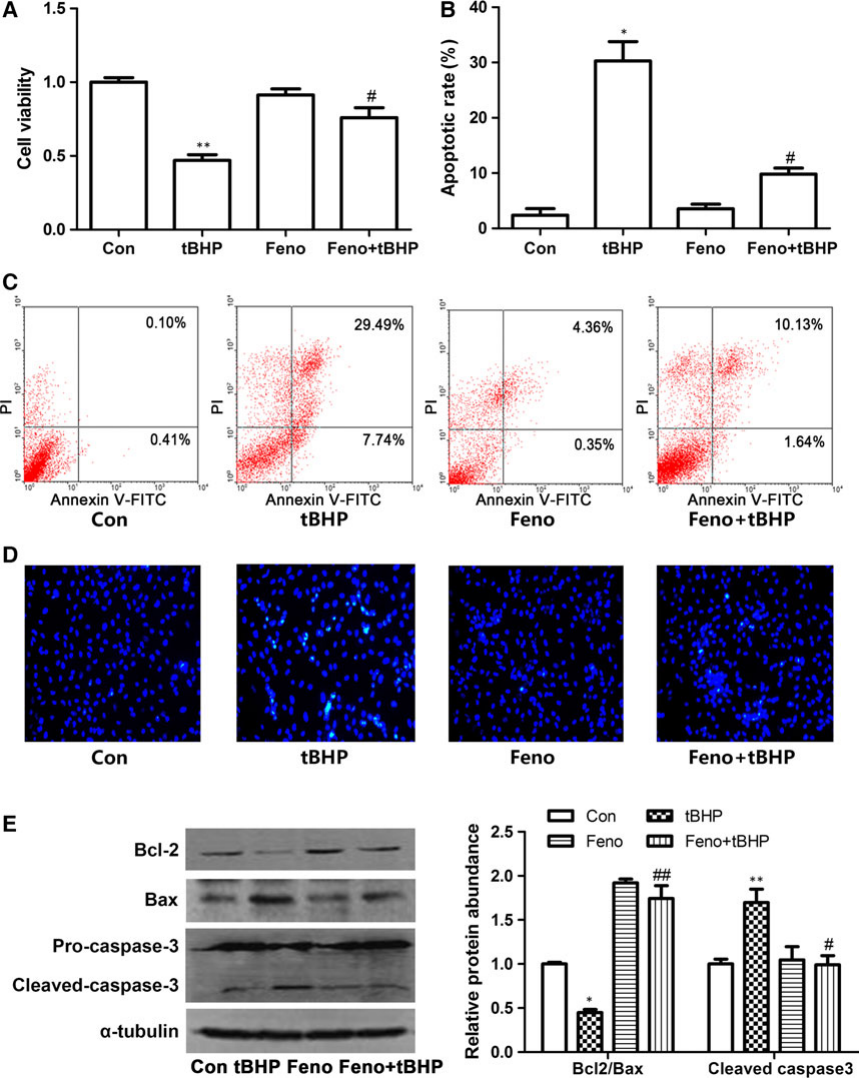
The protective effects of Feno on cardiomyocyte apoptosis induced by tBHP. (**A**) The decrease in cell viability were abrogated in tBHP group pre‐treated with Feno. (**B** and **C**) The cell apoptotic rate was increased significantly in tBHP group, however, decreased in cardiomyocytes pre‐treated with Feno. (**D**) Cell apoptosis was determined with Hoechst 33258 staining. 200× magnification. (**E**) Effects of Feno on the expression of apoptosis‐related proteins in tBHP‐induced cardiomyocytes. Mean ± S.D., *n* = 3, *P < 0.05, **P < 0.01 *versus* control, #*P* < 0.05, ##*P* < 0.01 *versus* tBHP.

**Figure 7:**
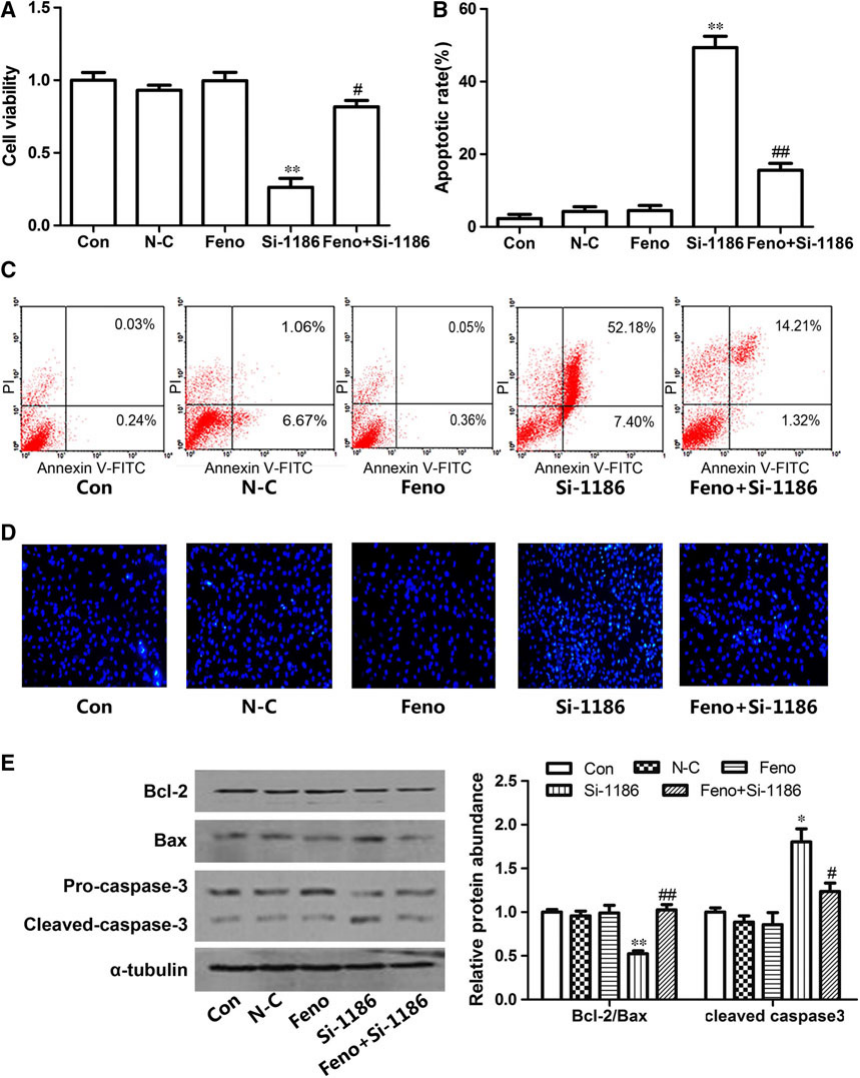
The protective effects of Feno on cardiomyocyte apoptosis induced by siRNA‐1186. (**A**) The derease of cell viability were abrogated in siRNA‐1186 group pre‐treated with Feno. (**B** and **C**) The cell apoptotic rate was increased significantly in siRNA‐1186 group, however, decreased in cardiomyocytes pre‐treated with Feno. (**D**) Cell apoptosis was determined with Hoechst 33258 staining. 200× magnification. (**E**) Effects of Feno on the expression of apoptosis‐related proteins in siRNA‐1186‐induced cardiomyocytes. N‐C: negative control siRNA. Mean ± S.D., *n* = 3, *P < 0.05, **P < 0.01 *versus* control, #*P* < 0.05, ##*P* < 0.01 *versus* si‐1186.

Peroxisome proliferator‐activated receptor α is a critical ligand‐activated transcription factor. To explore the possible mechanisms for the decrease of SCAD in cardiomyocyte apoptosis, the expression of PPARα was determined. Consistent with the changes of SCAD, PPARα protein and mRNA levels were markedly reduced in cardiomyocytes treated with 200 μM tBHP (Fig. 4A and B). In addition, we used fenofibrate (Feno, the PPARα agonist) to investigate the effect of PPARα activation on the expression of SCAD and cardiomyocyte apoptosis. Cardiomyocytes were pre‐treated with Feno (10 μM) for 30 min. and then treated by tBHP for 6 hrs or SCAD siRNA for 72 hrs. Compared with tBHP or SCAD siRNA groups, treatment with Feno significantly increased the expression and enzyme activity of SCAD, the uptake of free fatty acids and the ATP content (Figs 4C–E and 5A–E). Figures 6A and 7A showed that cell viability was significantly increased in cardiomyocyte pre‐treated with Feno compared with the tBHP or SCAD siRNA group. As the same time, Feno significantly decreased the cardiomyocyte apoptotic rate and the expression of cleaved caspase 3, but increased the ratio of Bcl‐2/Bax (Figs 6B–E and 7B–E). These findings indicated that cardiomyocyte apoptosis might be suppressed by the activation of PPARα and SCAD.

### Effects of AMPK on cardiomyocyte aoptosis

**Figure 8:**
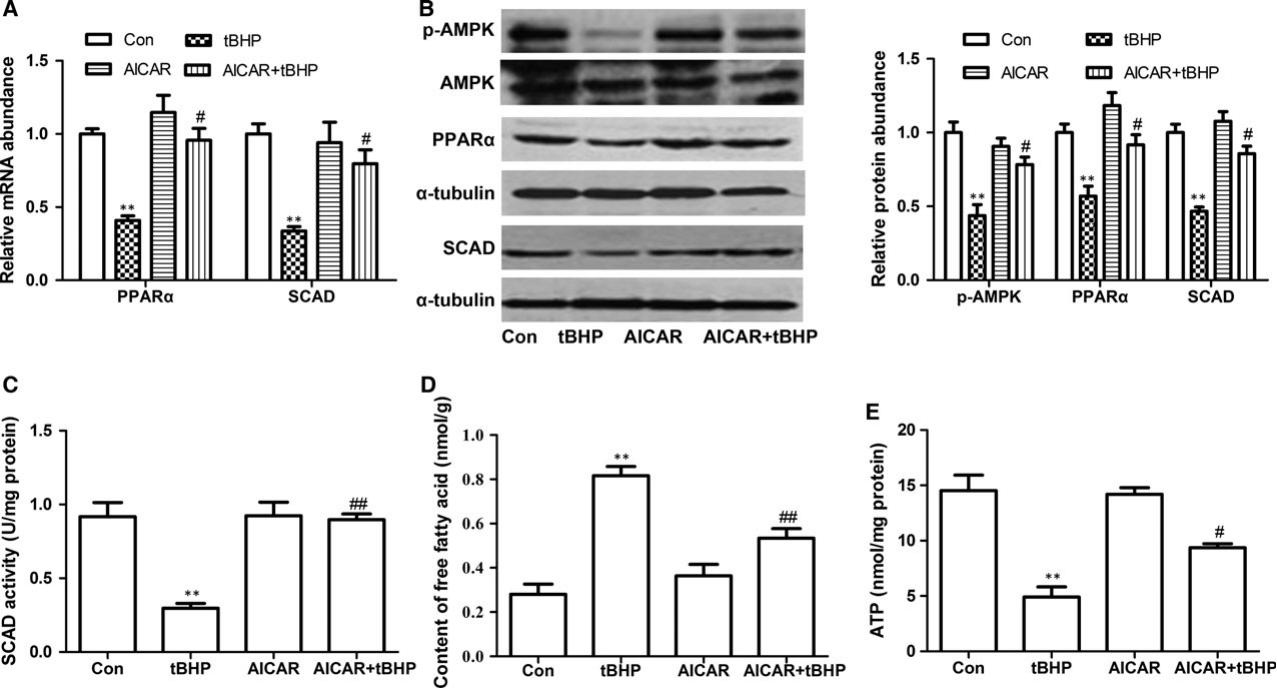
Effects of AICAR on cardiomyocyte apoptosis in tBHP‐treated cardiomyocytes. (**A**) The PPARα and SCAD mRNA expression was significantly decreased in cardiomyocytes treated with tBHP, however, increased in cardiomyocytes pre‐treated with AICAR. (**B**) The p‐AMPK, PPARα and SCAD protein expression was significantly decreased in cardiomyocytes treated with tBHP, however, increased in cardiomyocytes pre‐treated with AICAR. (**C**) The SCAD enzyme activity was significantly decreased in cardiomyocytes treated with tBHP, however, increased in cardiomyocytes pre‐treated with AICAR. (**D**) The content of free fatty acid was significantly increased in cardiomyocytes treated with tBHP, however, decreased in cardiomyocytes pre‐treated with AICAR. (**E**) The ATP level was significantly decreased in cardiomyocytes treated with tBHP, however, increased in cardiomyocytes pre‐treated with AICAR. Mean ± S.D., *n* = 3, *P < 0.05, **P < 0.01 *versus* control, #*P* < 0.05, ##*P* < 0.01 *versus* tBHP.

**Figure 9:**
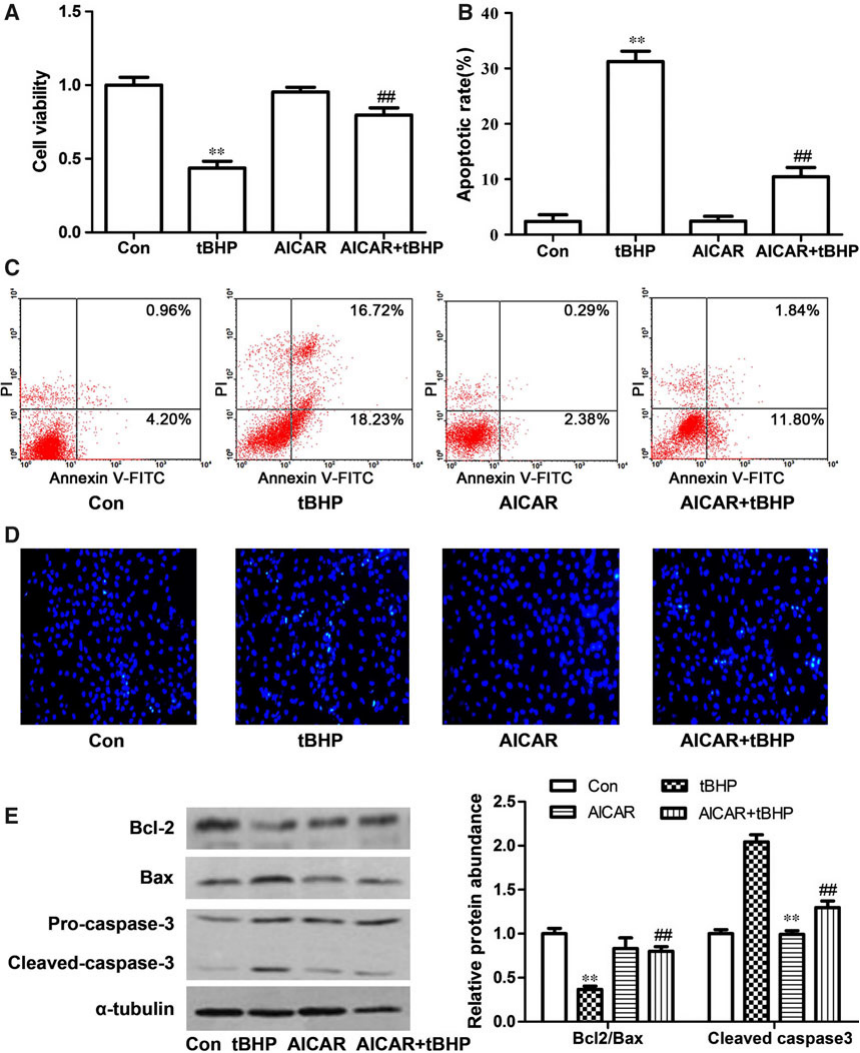
The protective effects of AICAR on cardiomyocyte apoptosis induced by tBHP. (**A**) The derease of cell viability were abrogated in tBHP group pre‐treated with AICAR. (**B** and **C**) The cell apoptotic rate was increased significantly in tBHP group, however, decreased in cardiomyocytes pre‐treated with AICAR. (**D**) Cell apoptosis was determined with Hoechst 33258 staining. 200× magnification. (**E**) Effects of AICAR on the expression of apoptosis‐related proteins in tBHP‐induced cardiomyocytes. Mean ± S.D., *n* = 3, *P < 0.05, **P < 0.01 *versus* control, #*P* < 0.05, ##*P* < 0.01 *versus* tBHP.

Previous study had demonstrated that the inhibition of AMPK by hydrogen peroxide was related to the cardiomyocytes apoptosis 19. To further investigate the possible mechanisms for the decrease of PPARα and SCAD in cardiomyocyte apoptosis, the expression of p‐AMPKα was determined. In the present study, we found that phosphorylation of AMPK at threonine‐172 (Thr‐172) site was decreased by 200 μM tBHP for 6 hrs. As shown in Figure 8A and B, the expression of p‐AMPKα, PPARα and SCAD were all significantly decreased in tBHP‐induced cardiomyocyte apoptosis. The enzyme activity of SCAD and ATP content were also decreased, however, the level of free fatty acid was increased in tBHP‐induced cardiomyocyte apoptosis (Fig. 8C–E). However, we pre‐treated cardiomyocytes with AMPK activator 5‐amino‐1‐β‐D‐ribofuranosyl‐imidazole‐4‐carboxamide (AICAR) (0.5 mM) for 30 min, then treated with tBHP for 6 hrs, these effects were all reversed by AICAR. In addition, compared with tBHP group, pre‐treatment with AICAR obviously increased the cell viability and the ratio of Bcl‐2/Bax, decreased the cardiomyocyte apoptotic rate and the expression of cleaved caspase 3 (Fig. 9A–E). These data demonsrated that cardiomyocyte apoptosis might be related with AMPK/PPARα/SCAD signal pathways.

## Discussion

Numerous researches have indicated that cardiomyocyte apoptosis plays a pivotal role in the pathogenesis of various cardiovasculr disease because of the loss of terminally differentiated cardiomyocytes 20. It is well‐known that myocardial is the one of high energy organ, and the chief energy of a normal adult heart is from fatty acid β‐oxidation 21, 22. Considerable evidence have supported the notion that the regulation of metabolic enzyme‐encoding genes in heart failure, especially the fatty acid oxidation enzyme 23, 24. In the present study, for the first time we show that SCAD expression and enzyme activity were significantly decreased in cardiomyocyte apoptosis. The present data have also investigated the molecular mechanism of SCAD in tBHP‐induced cardiomyocyte apoptosis.

Short‐chain acyl‐CoA dehydrogenase is the first enzyme of the short‐chain fatty acid β‐oxidation spiral. Our previous research indicated that SCAD played an important role in the pathological cardiac hypertrophy 10, 13. Otherwise, pathological cardiac hypertrophy will finally transit to heart failure, cardiomyocyte apoptosis is the important mechanism of myocardial hypertrophy transformation to heart failure. However, the role of SCAD in cardiomyocyte apoptosis remains unclear.

Tert‐butyl hydroperoxide, the similar character with H_2_O_2_, was used to imitate oxidative cell damage and apoptosis model. However, tBHP is more steability than H_2_O_2_. *In vitro*, it has been found to induce cell death in several cell lines such as human fibroblasts 25, hepatocytes 26 and primary cultures of cardiomyocytes 27, and cell apoptosis in low dose and cell death in high dose. In our research, we used various doses of tBHP for 6 hrs and 200 μM tBHP for different time. Results showed that tBHP could reduce the cell viability with some doses and time reliance. According to previous reports, the cell viability of 40–60% was used for apoptosis model 28. Therefore, 200 μM tBHP for 6 hrs was selected for subsequent experiments. In addition, we also found that the mRNA and protein level of SCAD was decreased with the increasing dose and time of tBHP. Of particular interest was that the expression of SCAD was decreased obviously by the 200 μM tBHP treated for 6 or 8 hrs. The changes of SCAD in cardiomyocyte apoptosis was consistent with that in pathological cardiac hypertrophy we previously reported 10, 13. These results indicated that SCAD may play an important role in cardiomyocyte apoptosis, which related with the transition of energy metabolism.

To determine the effect of SCAD in cardiomyocyte apoptosis, the technique of RNA interference was employed. SiRNA‐1186, the most efficient siRNA, significantly decreased the cell viability and the ratio of Bcl‐2/Bax, increased the cardiomyocyte apoptotic rate and the expression of cleaved caspase 3, which triggered the same apoptosis as cardiomyocytes treated with tBHP, which indicated that the down‐regulation of SCAD expression played an essential role in cardiomyocyte apoptosis. We also observed that the enzyme activity of SCAD, the uptake of free fatty acids and the ATP production were significantly reduced by siRNA 1186. The present data demonstrated that the down‐regulation of SCAD expression may result in the reduction of fatty acid β‐oxidation and cardiomyocyte apoptosis.

Peroxisome proliferator‐activated receptor α is highly expressed in the heart and which is known as a critical regulator of myocardial metabolism. It has been reported that the uptake of fatty acids and the protein expression of SCAD were lower in PPARα−/− mice than wild mice, which demonstrated that the expression of SCAD gene may be regulated by PPARα 26. Previously, we reported that the deactivation of PPARα led to the decrease of SCAD expression in pathological cardiac hypertrophy 10, 13. In the present study, we also found that the mRNA and protein expression of PPARα were markedly decreased in tBHP‐treated cardiomyocytes compared with the control group. The tendency were in keeping with the changes of SCAD. The specific PPARα ligand Feno significantly increased the expression and enzyme activity of SCAD, the uptake of free fatty acids and ATP production in tBHP‐ or siRNA‐1186‐treated cardiomyocytes. What is more, Feno significantly increased the cell viability and the ratio of Bcl‐2/Bax, decreased the cardiomyocyte apoptotic rate and the expression of cleaved caspase 3. These data demonstrated that the deactivation of PPARα may result in the decrease in SCAD expression in cardiomyocyte apoptosis.

AMP‐activated protein kinase, a well‐known energy sensor and regulator, is related to many cardiovascular diseases including cardiac ischaemia, myocardial infarction and heart failure 29. Previous studies demonstrated that the activation of AMPK provoked fatty oxidation through increased expression of PPARα in skeletal cells and cardiomyocytes 16, 30. Our results showed that the expression of p‐AMPKα was decreased in cardiomyocytes exposed to 200 μM tBHP for 6 hrs, which was in accordance with previous reports 31. The specific AMPK activator AICAR significantly increased the expression of PPARα and SCAD, the uptake of free fatty acids and ATP production in tBHP‐treated cardiomyocytes. In addition, AICAR significantly increased the cell viability and the ratio of Bcl‐2/Bax, decreased the cardiomyocyte apoptotic rate and the expression of cleaved caspase 3. These findings suggested that the dephosphorylation of AMPK induced the decreased expression of PPARα, leaded the decreased expression and enzyme activity of SCAD, which resulted in cardiomyocyte apoptosis.

## Conclusions

In summary, our study demonstrated that SCAD played an important role in cardiomyocyte apoptosis, which could be regulated by AMPK/PPARα/SCAD signal pathway. These novel findings demonstrated that SCAD may be as a new target to prevent the development of heart failure.

## Conflicts of interest

No authors have conflicts of interest to declare.
